# Left atrial function index predicts poor outcomes in acute myocardial infarction patients treated with percutaneous coronary intervention

**DOI:** 10.3389/fcvm.2023.1043775

**Published:** 2023-09-01

**Authors:** Yijin Tang, Siling Peng, Hui-ling Yao, Zhibin Liu, Liang Zhang, Changqing Zhong, Chang She, Wei Liu, Yi Tang, Qinghua Fu, Yi Zhang

**Affiliations:** ^1^ Department of Cardiology, Hunan Provincial People’s Hospital, The First Affiliated Hospital of Hunan Normal University, Changsha, China; ^2^ Department of General Medicine, Hunan Provincial People’s Hospital, The First Affiliated Hospital of Hunan Normal University, Hunan Normal University, Changsha, China; ^3^ Department of Cardiology, Hunan Provincial People's Hospital, The First Affiliated Hospital of Hunan Normal University, Clinical Medicine Research Center of Heart Failure of Hunan Province, Hunan Normal University, Changsha, China

**Keywords:** left atrial function index, acute myocardial infarction, transthoracic echocardiography, percutaneous coronary intervention, prognosis

## Abstract

**Background and aims:**

The left atrial function index (LAFI) is an index that combines the left atrial emptying fraction, adjusted left atrial volume and stroke volume. The prognostic value of LAFI in acute myocardial infarction (AMI) patients treated with percutaneous coronary intervention (PCI) is unknown. This study aims to determine whether LAFI predicts prognosis in AMI patients treated with PCI.

**Methods:**

Patients with newly diagnosed AMI who were treated with PCI at Hunan Provincial People's Hospital from March 2020 to October 2021 were prospectively enrolled. All patients underwent transthoracic echocardiography (TTE) at baseline and follow-up. The endpoint events included rehospitalization due to unstable angina, nonfatal myocardial infarction, rehospitalization due to heart failure and cardiovascular death.

**Results:**

A total of 368 patients with AMI (92 women; mean age, 61.45 ± 11.91 years) were studied with a median follow-up of 14 ± 6.58 months. Sixty-nine patients had endpoint events. Patients who presented with events had a significantly lower LAFI than patients without events (34.25 ± 12.86 vs. 48.38 ± 19.42, *P* < 0.0001). Multivariate Cox analysis demonstrated that LAFI (HR = 0.97 [95% CI: 0.95; 0.99]; *P* = 0.012) and the Killip classification (HR = 1.51 [95% CI: 1.03; 2.22]; *P* = 0.034) were independently predictive of endpoint events. Kaplan–Meier survival curves showed that patients with LAFI ≤ 40.17 cm/ml/m^2^ had higher events than patients with LAFI > 40.17 cm/ml/m^2^ (HR = 8.53 [95% CI: 4.74; 15.35]; *P* < 0.0001).

**Conclusion:**

LAFI is a strong and independent predictor of adverse events and can be used for risk stratification in patients with AMI treated with PCI.

## Introduction

Acute myocardial infarction (AMI) remains a leading cause of mortality worldwide, despite substantial improvements in prognosis over the past decade (1). However, some patients still experience adverse events, such as unstable angina, nonfatal myocardial infarction, heart failure after myocardial infarction and death even after receiving percutaneous coronary intervention (PCI). This places a major economic and resource burden on the public health system (2). Therefore, it is important to identify patients with a higher risk of adverse events after AMI in order to treat these patients with intensive drugs at the early stage to improve their prognosis. Transthoracic echocardiography (TTE) is a noninvasive, low-cost, and easily available bedside imaging tool that detects the motion of the myocardial walls, damage extent, functional consequences, and mechanical complications; therefore, TEE is widely used for risk stratification in patients with AMI (3). The left ventricular ejection fraction (LVEF) obtained from echocardiography is often used to assess left ventricular (LV) systolic dysfunction, which can predict poor outcome in patients with AMI. However, LVEF only reflects LV systolic dysfunction, which cannot reveal LV diastolic dysfunction, left atrial (LA) volume, as well as LA function. Meanwhile, several studies have demonstrated that the LA volume index and LA emptying fraction (LAEF), which reflect LA volume and LA function, respectively, could predict morbidity or mortality after AMI (4–6). However, these parameters cannot reflect LV systolic dysfunction. Researchers have attempted to find a better parameter that can reflect both LV systolic and diastolic function, as well as LA function, to predict prognosis in patients with AMI.

The left atrial function index (LAFI) was such a parameter, initially proposed by Liza et al. in 2008 (7) and it is calculated as LAFI = [LAEF × LV outflow tract-velocity time integral (LVOT-VTI)]/LA end-systolic volume index (LAESVi). The LAFI incorporates analogues of cardiac output, atrial reservoir function and LA size, which reflects LV systolic and diastolic function, as well as LA function. Previous studies showed that LAFI was a good predictor of hospitalization for heart failure in patients with preserved ejection fraction and coronary heart disease, and could also predict long-term survival in stable outpatients with systolic heart failure (8, 9). However, whether LAFI could be used to predict the prognosis of patients with AMI treated with PCI is unknown. This study intended to explore the value of the LAFI in the prognostic evaluation of patients with AMI treated with PCI.

## Methods

### Study population

Patients who were diagnosed with AMI and received PCI in hospital at Hunan Provincial People's Hospital between March 2020 and October 2021 were enrolled. The diagnostic criteria for AMI, including ST segment elevation myocardial infarction (STEMI) and non-ST segment elevation myocardial infarction (NSTEMI), was based on clinical guidelines (10). Patients who only underwent culprit-lesion PCI all came to our hospital underwent the second PCI for complete revascularization after 1 month. Patients with absent or poor imaging of the atrium and moderate to severe degrees of mitral regurgitation were excluded. This research was conducted in compliance with the Declaration of Helsinki and was approved by the Ethics Committee of Hunan Provincial People's Hospital. Informed consent was obtained from all enrolled patients.

### Echocardiographic methods

We performed resting TTE (GE Vivid E9, America) for all patients within 2 days after they underwent PCI. TTE was performed in the standard left lateral recumbent and supine positions. Routine M-mode and 2-dimensional echocardiography were performed using a standard protocol (11). The maximum LA volume (LAmax) and minimum LA volume (LAmin) were determined by averaging LAmax and LAmin measurements from the apical two- and four-chamber views using the recommended Simpson's biplane summation of disks method. LAEF was calculated as [(LAmax–LAmin)/LAmax] × 100%. The LAESVi was calculated by dividing LA end-systolic volumes by body surface area. LV end-diastolic (LVEDV) and LV end-systolic volumes (LVESV) were measured using Simpson's method in the apical-4 chamber and the apical-2 chamber view. Stroke volume was calculated as (LVEDV-LVESV), and LVEF was calculated as (Stroke volume/LVEDV) × 100%. LVOT-VTI was measured by manually tracing pulsed Doppler velocities in the LV outflow tract in apical 5-chamber views. The final measures were derived by averaging the measurements over ≥3 cardiac cycles. The LAFI was calculated using a previously validated formula: LAFI = (LAEF × LVOT-VTI)/LAESVi (7).

### Clinical assessment and follow-up

Basic demographic data, biochemical tests, Killip classification and coronary arteriography were collected at baseline. All enrolled patients were followed up telephonically at 1, 3, 6, 12, and 18 months after discharge, and the endpoint events during this period were recorded. The endpoint events were defined as rehospitalization due to unstable angina, nonfatal myocardial infarction, rehospitalization due to heart failure and cardiovascular death. The follow-up ended on May 1, 2022. The first occurrence of the event, rather than a cumulative event, was taken into consideration in our analysis.

### Statistical method

Continuous variables with a normal distribution are expressed as the mean ± standard deviation ($\bar{x}\pm s$), and continuous variables with a nonnormal distribution are represented by the median and quartile (IQR). One-way analysis of variance (ANOVA), Student's *t*-test or Mann-Whitney *U* test was used for comparison as appropriate. The categorical variables are expressed as *n* (%), and the Chi-square (*χ*^2^) test was used for categorical variables. Pearson or Spearman correlation coefficients were used for bivariate correlation analysis. Receiver Operating Characteristic (ROC) Curve was used to judge the performance of variables in prognostic prediction and to determine the best cut-off point. Univariate and multivariate Cox proportional hazards model and Kaplan-Meier curve were used for survival analysis. The method of “enter” was used in the multivariable Cox analysis. The ROC curve was analysed using MedCalc v19.3.0, and the rest of the assays were analysed using SPSS 23.0. Two-tailed *P* value <0.05 was statistically significant.

## Results

### Baseline characteristics and follow-up

**Figure 1:**
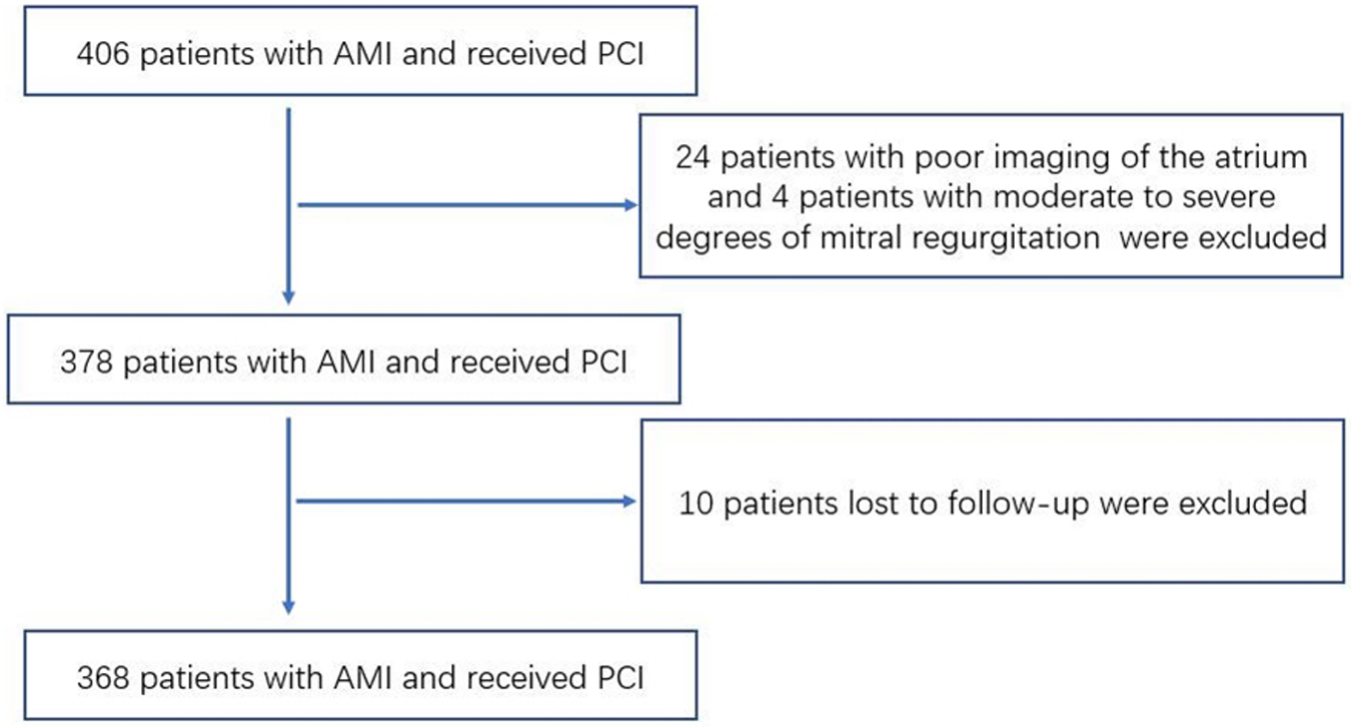
Patients’ flow-chart in our study.

Initially, a total of 406 patients were enrolled in our study. Of those, 24 patients had poor imaging of the atrium, 4 patients had moderate to severe degrees of mitral regurgitation and 10 patients lost to follow-up were not included in the analysis. The remaining 368 patients were included in the final analysis of our study (Figure 1). The mean age was 61.45 ± 11.91 years, and 25.0% of patients were women. The median follow-up time was 14.76 ± 6.58 months, and 69 patients developed events during the follow-up period, including 13 patients readmitted due to unstable angina pectoris, 31 patients readmitted due to heart failure, 11 patients with nonfatal myocardial infarction, and 14 patients with cardiovascular death.

### Differences in variables between groups

Patients with events had a similar sex distribution and body mass index compared with patients without events (Non-events). However, patients with adverse events are much older, had a higher proportion of type 2 diabetes mellitus (T2DM) and multivessel coronary artery disease (MVD), a poorer Killip classification, higher levels of N-terminal fragment of pro B-type natriuretic peptide (NT-proBNP) and white blood cell count (WBC) compared to patients with non-events. In terms of echocardiography parameters, patients who presented with adverse events had significantly lower LAEF, LAFI, LVEF, and LVOT-VTI and higher LAESVi and LVEDV. In addition, a higher proportion of patients with events were treated with diuretics (Table 1).

**Table 1:** Comparison of baseline data between patients with or without events.

Variables	Events (*n* = 69)	Non-events (*n* = 299)	*P*
Clinical characteristics			
Male, *n* (%)	48 (69.6%)	228 (76.3%)	NS
Age, year	65.42 ± 11.36	60.54 ± 11.87	0.001
BMI, (kg/cm^2^)	24.17 ± 3.66	24.33 ± 3.43	NS
Smoking, *n* (%)	41 (59.4%)	206 (68.9%)	NS
Hypertension, *n* (%)	39 (56.5%)	173 (57.9%)	NS
T2DM, *n* (%)	27 (39.1%)	80 (26.8%)	0.042
Previous CI, *n* (%)	8 (11.6%)	29 (9.7%)	NS
Previous MI, *n* (%)	12 (17.4%)	30 (10.1%)	NS
Atrial fibrillation, *n* (%)	2 (2.9%)	5 (1.7%)	NS
The Killip classification, *n* (%)			0.001
I/II	59 (85.5%)	287 (96.0%)	
III/IV	10 (14.5%)	12 (4.0%)	
Biochemical parameters			
WBC, ×10^9^/L	10.22 ± 3.98	8.76 ± 2.71	0.012
TC, mmol/L	4.46 ± 1.21	5.16 ± 13.00	NS
LDL, mmol/L	2.69 ± 0.91	2.62 ± 0.96	NS
eGFR, ml/min/1.73m^2^	88.22 ± 42.07	93.97 ± 35.70	NS
TB, umol/L	13.29 ± 6.51	14.75 ± 21.10	NS
NT-proBNP, ng/L	6,094.52 ± 8,140.09	2,088.71 ± 4,120.15	<0.0001
Coronary arteriography			
Culprit vessel, *n* (%)			0.85
LAD	39 (56.5%)	172 (57.5%)	
LCX	9 (13.0%)	32 (10.7%)	
RCA	21 (30.4%)	95 (31.7%)	
MVD, *n* (%)	66 (95.7%)	240 (80.3%)	0.002
Echocardiography			
LAESVi, ml/m^2^	28.85 ± 8.15	25.56 ± 8.61	<0.001
LAEF, %	50.69 ± 8.39	56.88 ± 10.57	<0.0001
LVOT-VTI, cm	18.18 ± 3.79	19.68 ± 4.19	<0.007
LAFI, cm/ml/m^2^	34.25 ± 12.86	48.38 ± 19.42	<0.0001
LVEF, %	44.00 ± 10.29	52.93 ± 10.00	<0.0001
LVEDV, ml	82.72 ± 21.82	77.09 ± 27.47	0.004
Therapeutics			
Aspirin, *n* (%)	68 (98.6%)	297 (99.3%)	NS
P2Y12 inhibitor (clopidogrel or ticagrelor), *n* (%)	69 (100%)	297 (99.3%)	NS
β-blocker, *n* (%)	61 (88.4%)	276 (92.3%)	NS
ACEI/ARB, *n* (%)	60 (87.0%)	263 (88.0%)	NS
Statin, *n* (%)	69 (100%)	296 (99.0%)	NS
Diuretics, *n* (%)	10 (14.5%)	18 (6.0%)	0.017

Continuous data are mean ± standard deviation or median (interquartile range), and categorical variables are *n* (%). ACEI/ARB, angiotensin-converting enzyme inhibit or angiotensin receptor blocker; BMI, body mass index; CI, cerebral infarction; eGFR, estimated glomerular filtration rate; LAD, left anterior descending artery; LAEF, left atrial emptying fraction; LAESVi, left atrial end-systolic volume index; LAFI, left atrial function index; LCX, left circumflex artery; LDL, low density lipoprotein; LVOT-VTI, the left ventricular outflow tract velocity time integral; LVEF, left ventricular ejection fraction; LVEDV, left ventricular end-diastolic volume; MVD, multi-vessel coronary artery disease; NT-proBNP, NT-terminal B-type brain natriuretic peptide precursor; MI, myocardial infarction; RCA, right coronary artery; TC, total cholesterol; TB, total bilirubin;T2DM, type 2 diabetes mellitus; WBC, white blood cell count.

### Correlation analysis

**Figure 2:**
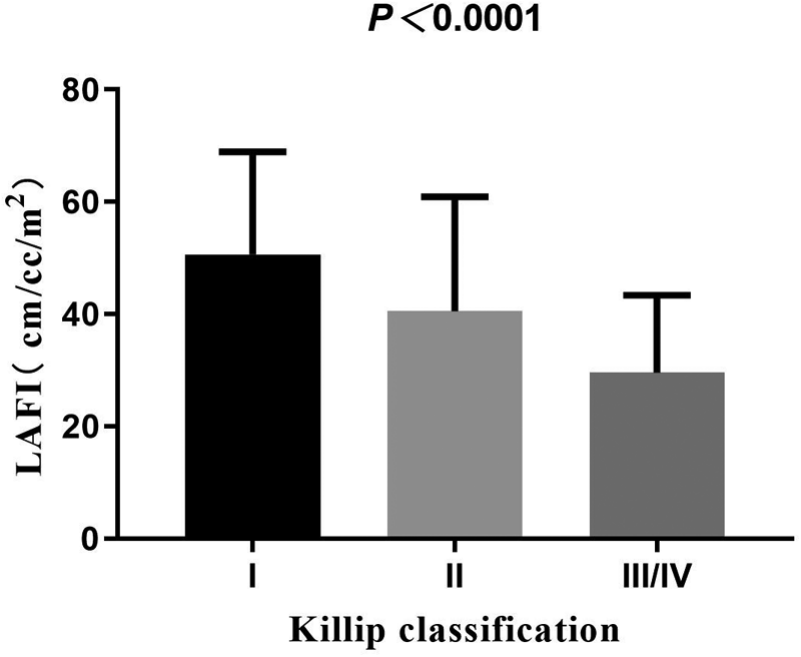
LAFI with Killip classification.

With the increase in Killip classification, the levels of LAFI were decreased (Figure 2). In our study, 7 patients had atrial fibrillation and 361 patients did not have atrial fibrillation. Compared to patients without atrial fibrillation, LAFI levels were significantly lower in patients with atrial fibrillation (29.47 ± 15.04 vs. 46.05 ± 19.12 cm/ml/m^2^, *P* = 0.02). Correlation analysis showed that LAFI levels correlated positively with LVEF and estimated glomerular filtration rate (eGFR) (*r* = 0.62, 0.24, all *P* < 0.001) and negatively with age, NT-proBNP, and LVEDV (*r* = −0.21, −0.50, −0.48, all *P* < 0.001).

### Prediction of the composite outcome

LAFI had the highest area under the receiver operator characteristic curve (AUC) value in predicting the events when compared with its individual components LAEF, LAESVi and LVOT-VTI (C-statistics: LAFI 0.73 > LAEF 0.70 > LAESVi 0.64 > LVOT-VTI 0.59). The calculated optimal point of LAFI was 40.17 cm/ml/m^2^. The sensitivity and specificity for predicting the events were 78.26% and 66.56%, respectively. The AUC and the calculated optimal point of LAFI and other important echocardiography parameters were shown in Table 2. Multivariable Cox models, which included LAFI and its components, also revealed that LAFI provided prognostic value incremental to its individual components (Table 3).

**Table 2:** The ROC analysis of important echocardiography parameters.

Variables	AUC (95% CI)	Sensitivity/specificity	Cut-off value
LAEF	0.70 (0.66, 0.75)	66.67%/71.57%	≤52%
LAESVi	0.64 (0.58, 0.68)	63.77%/62.21%	>26.10 ml/m^2^
LVOT-VTI	0.59 (0.54, 0.64)	69.13%/78.26%	≤16.4 cm
LAFI	0.73 (0.70, 0.77)	78.26%/66.56%	≤40.17 cm/ml/m^2^
LVEDV	0.61 (0.56, 0.66)	60.87%/63.88%	>77.32 ml
LVEF	0.74 (0.69, 0.78)	68.12%/69.90%	≤48%

LAEF, left atrial emptying fraction; LAESVi, left atrial end-systolic volume index; LAFI, left atrial function index; LVOT-VTI, the left ventricular outflow tract velocity time integral; LVEF, left ventricular ejection fraction; LVEDV, left ventricular end-diastolic volume.

**Table 3:** Cox analysis of LAFI and its components.

Variables	Wald	HR	95% CI	*P*-value
LAFI	13.67	0.89	0.83–0.94	<0.0001
LAEF	3.75	1.05	1.00–1.10	0.053
LVOT-VTI	3.86	1.14	1.00–1.29	0.050
LAESVi	5.56	0.92	0.86–0.99	0.018

LAEF, left atrial emptying fraction; LAESVi, left atrial end-systolic volume index; LAFI, left atrial function index; LVOT-VTI, the left ventricular outflow tract velocity time integral.

On univariate Cox regression analysis, age, T2DM, NT-proBNP, MVD, Killip classification, and variables obtained from echocardiography (LAESVi, LAEF, LVOT-VTI, LAFI, LVEF, LVEDV) were significant predictors of events. Since there were not enough events and LAESVi, LAEF, LVOT-VTI and LAFI exhibited collinearity, we only included age, T2DM, NT-proBNP, Killip Classification, MVD, LAFI, LVEF, LVEDV and diuretics in the multivariable Cox analysis. The results showed that Killip classification (HR = 1.51 [95% CI: 1.03; 2.22]; *P* = 0.034) and LAFI (HR = 0.97 [95% CI: 0.95; 0.99]; *P* = 0.012) were independent predictors of events (Table 4).

**Table 4:** Cox analysis of proportional risks for events.

Variables	Univariate			Multivariate		
	HR	95% CI	*P*-value	HR	95% CI	*P*-value
Age	1.03	1.01–1.05	0.004	1.01	0.98–1.03	0.613
T2DM	1.73	1.07–2.81	0.026	1.30	0.78–2.16	0.317
The Killip classification	2.28	1.72–3.02	<0.0001	1.51	1.03–2.22	0.034
NT-proBNP	1.00	1.00–1.00	<0.0001	1.00	1.00–1.00	0.277
MVD	5.47	1.72–17.41	0.004	2.70	0.82–8.81	0.101
LAFI	0.95	0.94–0.97	<0.0001	0.97	0.95–0.99	0.012
LVEF	0.93	0.91–0.95	<0.0001	0.97	0.94–1.01	0.112
LVEDV	1.01	1.00–1.02	0.03	0.99	0.98–1.00	0.145
Diuretics	2.40	1.22–4.69	0.011	1.22	0.56–2.62	0.617

HR, hazard ratio; CI, confidence interval; T2DM, type 2 diabetes mellitus; NT-proBNP, NT-terminal B-type brain natriuretic peptide precursor; MVD, multi-vessel coronary artery disease; LAFI, left atrial function index; LVEF, left ventricular ejection fraction; LVEDV, left ventricular end-diastolic volume.

**Figure 3:**
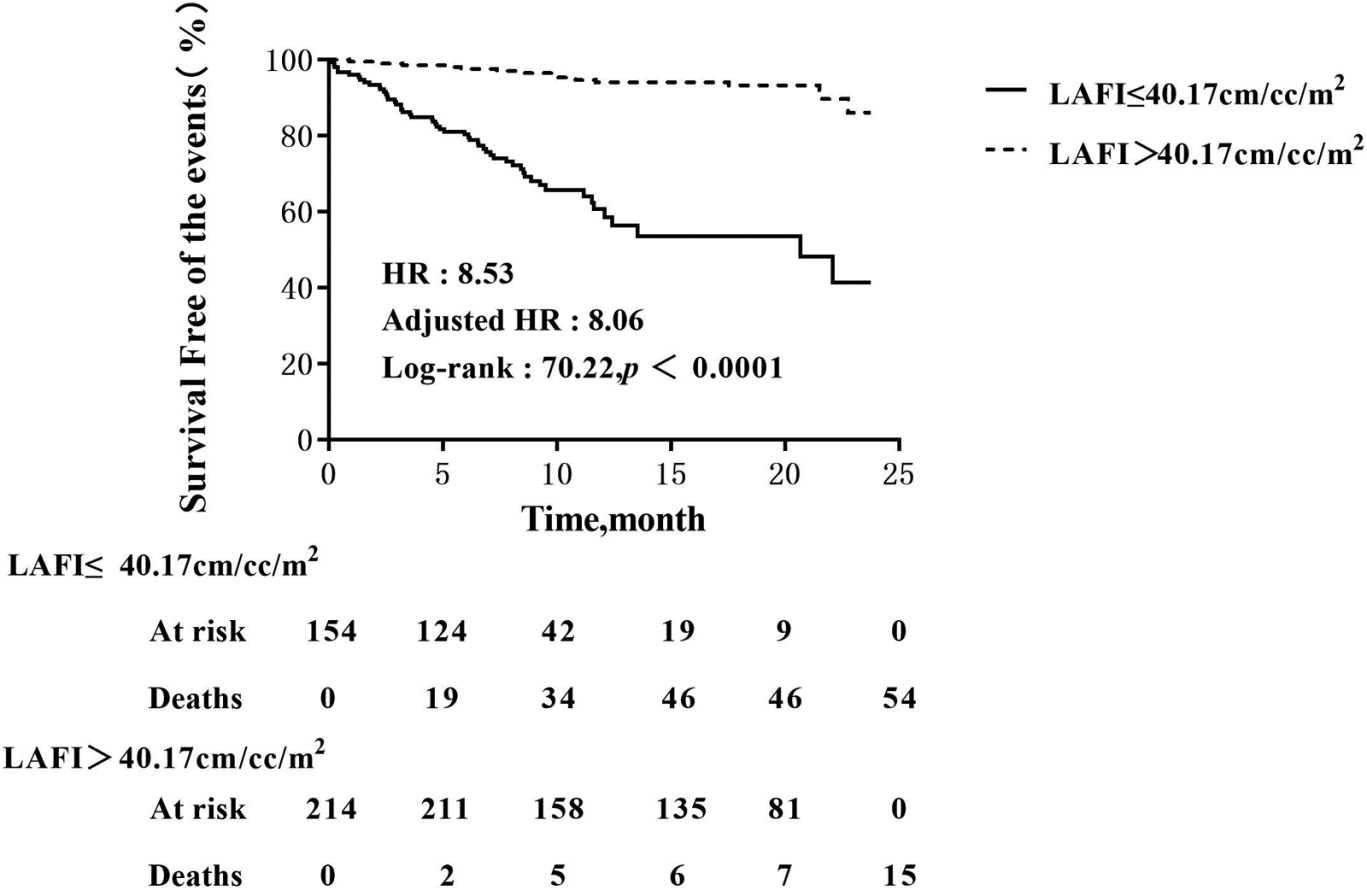
Kaplan–Meier analysis of LAFI for events. Adjusted HR indicates hazard ratio (HR) adjusted for age; LAFI, left atrial function index.

Patients with LAFI ≤ 40.17 cm/ml/m^2^ had a worse survival rate than patients with LAFI > 40.17 cm/ml/m^2^. Kaplan‒Meier survival estimates (Figure 3) showed early separation of the event-free survival curves, which continued to diverge throughout follow-up. The unadjusted HR was 8.53 ([95% CI: 4.74; 15.35]; *P* < 0.0001), and after adjustment for age, the HR was 8.06 ([95% CI: 4.46; 14.56]; *P* < 0.0001).

## Discussion

We first evaluated the prognostic value of the LAFI in a cohort of 368 AMI patients treated with PCI. We found that LAFI was negatively correlated with NT-proBNP and positively correlated with LVEF; furthermore, the results showed that patients with LAFI were associated with poor prognosis. Importantly, the prognostic value of the LAFI was independent of a wide range of clinical risk factors and laboratory and echocardiographic parameters.

LAEF is an indicator of functional LA remodelling, and LAESVi reflects LA structural remodelling. A previous study found that LAEF had a weak correlation with LAESVi (12). By incorporating both LAEF and LAESVi in one formula, LAFI is a more comprehensive indicator of LA remodelling (13). Rigatelli G et al. showed that LAFI was an useful marker of atrial dysfunction severity in patients with patent foramen ovale before and after the interventional procedure (14). LA remodelling can promote the occurrence of atrial fibrillation, and Sardana et al. demonstrated that LAFI, an indicator of LA remodeling, was associated with incidental atrial fibrillation in Framingham Offspring Study participants (15). Meanwhile, atrial fibrillation can decrease LA contractile function and lead to enlargement of the LA, and Nagase et al. also demonstrated that catheter ablation could improve LAFI in patients with atrial fibrillation (16). The results of our study showed that the LAFI was lower in subjects with atrial fibrillation than in subjects without atrial fibrillation, which are consistent with the results of previous studies (8, 9).

LAFI combines not only LAEF and LAESVi but also LVOT-VTI. In other words, it not only reflects LA structure and function, but also reflects both LV systolic and diastolic function (17). Therefore, LAFI may provide greater prognostic information than a single parameter, such as LVEF, LAESVi or LAEF. Studies have demonstrated that LAFI, superior to other echocardiography parameters, could predict long-term survival in stable systolic heart failure outpatients with LVEF < 40% and patients with preserved ejection fraction and coronary heart disease (8, 9). Shamekhi et al. reported that transcatheter aortic valve replacement (TAVR) could improve LAFI within 12 months after the procedure and a reduced LAFI was an independent predictor of mortality in patients with severe aortic stenosis (18). In addition, Sardana et al. found that LAFI was associated with atrial fibrillation recurrence after catheter ablation in patients with atrial fibrillation (19). The results of our study showed that LAFI had a positive association with LVEF, an indicator of positive cardiac remodelling, and an inverse association with LVEDV, an indicator of adverse cardiac remodelling. Although the AUC value of LVEF was slightly higher than LAFI. However, LAFI showed better performance than LVEF in multivariable Cox analysis. In addition, LAFI, rather than LVEF, could independently predict the events after adjusting for significant confounders, which was consistent with the results of the studies that we mentioned above (8, 9, 18, 19).

In our study, patients with LAFI ≤ 40.17 cm/ml/m^2^ had a worse survival rate than patients with LAFI > 40.17 cm/ml/m^2^, which supported that LAFI was useful in the risk stratification of patients with AMI with PCI. Shamekhi et al. found that severe aortic stenosis patients with a LAFI ≤ 13.5 cm/ml/m^2^ showed significantly higher rate of 1-year mortality, compared to those with a LAFI > 13.5 cm/ml/m^2^ (18). Sargento et al. reported that heart failure with reduced ejection fraction patients with LAFI < 16.57 cm/ml/m^2^ had a worse adverse outcomes than patients with LAFI ≥ 16.57 cm/ml/m^2^ (9). The calculated optimal point of LAFI for predicting the events in our study is different from that in other studies (9, 18). We speculate that the reason is mainly attributed to different study populations, as the LAFI of patients with severe aortic stenosis or reduced ejection fraction heart failure was usually lower than patients with AMI with PCI.

### Study limitations

There are several limitations to our study. First, we did not measure LAFI before patients underwent PCI and the day when the patient was discharged. Second, as there was a lack of electrocardiogram recording, we did not record the incidence of atrial fibrillation. Third, our study was a single-center study, and the sample size was relatively small. A multi-center study with a large-scale sample will be required to further validate these results.

## Conclusions

LAFI is a strong and independent predictor of events and can be used for risk stratification in patients with AMI treated with PCI.

## Data Availability

The raw data supporting the conclusions of this article will be made available by the authors, without undue reservation.
